# Resources and facilitators of workplace well-being among healthcare professionals in a hospital setting—results of a qualitative interview study

**DOI:** 10.3389/fpubh.2025.1586976

**Published:** 2025-05-26

**Authors:** Julia Berschick, Judith Czakert, Anna Katharina Koch, Marleen Schröter, Melanie Steinmetz, Martin Bogdanski, Julia Katharina Schiele, Christian Kessler, Georg Seifert, Wiebke Stritter

**Affiliations:** ^1^Department of Pediatric Oncology and Hematology, Charité – Universitätsmedizin Berlin, Corporate Member of Freie Universität Berlin and Humboldt Universität zu Berlin, Berlin, Germany; ^2^Charité Competence Center for Traditional and Integrative Medicine (CCCTIM), Charité – Universitätsmedizin Berlin, Corporate Member of Freie Universität Berlin and Humboldt Universität zu Berlin, Berlin, Germany; ^3^Institute of Social Medicine, Epidemiology and Health Economics, Charité – Universitätsmedizin Berlin, Corporate Member of Freie Universität Berlin and Humboldt Universität zu Berlin, Berlin, Germany; ^4^Department of Internal and Integrative Medicine, Immanuel Krankenhaus Berlin, Berlin, Germany

**Keywords:** healthcare professionals, workplace health promotion, resources, burnout, stress, resilience, integrative medicine, mind–body-medicine

## Abstract

**Background:**

Working in a hospital setting can be rewarding but also represents a demanding and often stressful work environment due to personnel shortage and high work volumes among others. A considerable body of literature addresses the adverse effects of working conditions that often result in poor well-being of medical professionals. This work moves from a problem-centered approach towards resilience-focused pathways. It does so by focusing on (self-reported) individual and organizational resources and facilitators of workplace well-being through medical professionals’ ability to perceive and engage with capacities within a demanding work environment.

**Methods:**

This paper is based on a qualitative study in which data was collected in two different German hospitals through interviews with medical doctors, nurses and medical assistants. Data was analyzed through inductive-deductive qualitative content analysis.

**Results:**

Our findings show a variety of resources in 4 domains overarching individual solution-oriented mindset constitutions, success-driven behaviors, sense of meaning as well as resource-enhancing environmental factors. Healthcare professionals show diverse individual strategies and behavioral patterns to build resilience and foster proactive approaches to deal with challenges and high-pressure situations.

**Conclusion:**

Beneficial influencing factors could be identified that reveal underlying processes and opportunities to prevent negative health-related outcomes. The findings provide valuable insights into specific individual coping strategies and attitudes that appear to be associated with resourcefulness. Information is provided to institutionally and individually support a successful management of a health professionals’ work life.

## 1 Background

The quality and sustainability of healthcare systems largely rely on the healthcare professionals who deliver care (1). Effective healthcare, as emphasized by person-centered medicine, is best achieved when it involves providers equally as recipients (2). However, healthcare professionals face a variety of challenges such as high demands and low control and they often report distinct job-dissatisfaction (3–5). Mounting pressures of government regulations as well as the business dimensions of medicine have already been addressed decades ago and place a significant added systemic burden on individuals (6). A considerable body of literature focusses on the adverse effects of working conditions of medical professionals resulting in poor well-being, burnout and compassion fatigue (7–9). The COVID-19 pandemic has highlighted the necessity of a well-functioning healthcare system as well as the importance of maintaining healthy and resilient healthcare professionals (3, 10). While ongoing efforts to implement structural changes are crucial, substantial endeavors are still necessary to improve the situation, as advocated by the World Health Organization (WHO) (11, 12). Meeting these needs requires a concerted effort to consolidate all available forces, including resources and potential of personnel themselves (11, 12). Recent research has also underscored the necessity of fundamental changes, also addressing the significance of resilience and its development in this context (1, 13). Resilience and resources are interconnected yet distinct concepts (14, 15). Whereas resilience refers to the capacity to adapt, recover, and bounce back from stress, adversity or challenges, resources refer to the assets, tools, or support systems available to individuals that enable them to achieve goals, cope with stress, or perform tasks effectively (16). Resilience therefore is more about the ability to respond to challenges, while resources are the means or assets that support this ability (17). The conceptualization of resilience as either a process, a trait or an outcome underscores it’s complexity (18). Furthermore, while it may be appealing to adopt a binary view of resilience as either present or absent, it is more accurately understood as existing along a continuum with varying degrees across different areas of life (18, 19). For instance, an individual may exhibit strong adaptive abilities in professional environments while facing challenges in personal relationships or other aspects of life (18). Moreover, environmental, temporal as well as inter-social elements have been pointed out as enhancing or limiting factors to resilience emphasizing the importance of examining not only the individual but also the broader context in which they operate (18). In terms of characteristics, numerous personality traits and behaviors have been identified as contributing to the promotion of resilience though our ability to reliably determine who will exhibit resilience is limited (20). The nature of this resilience paradox directs our attention towards the resources, individuals access within this high-demand work setting (20). Drawing on the *Job Demands-Resources theory* by Bakker and De Vries, which highlights how job strain in such settings fosters avoidance coping and subsequent resource depletion, we aim to explore the attitudes individuals adopt to mitigate resource loss and maintain their capacity for effective action (21). These attitudes may further illuminate the motivational underpinnings and the role of mindset in shaping medical professional’s ability to navigate adversity (22). Given the limitations associated with the validity of many questionnaire-based studies, employing semi-structured interviews seems to represent a critical methodological approach to generating robust and nuanced insights in this domain (23–25).

As research of resources among healthcare professionals as well as the specific attitudes that facilitate a strong and stable mindset, enabling a proactive and effective approach to dealing with daily work-related demands is promising, it has received little attention in research so far (26, 27).

### 1.1 Aims and research question

The focus of interest is on the adaptational abilities of healthcare professionals to cultivate resourcefulness in response to (work-related) challenges, reframing those obstacles as opportunities for personal growth.

The underlying research questions are:

What insights can be gained regarding the resources of healthcare professionals working in hospitals to cope with the demands of their work environment?

Which implications can be drawn from the results for future programs for the promotion of workplace well-being?

### 1.2 Involvement in research

This paper is published as part of the ongoing project LAGOM.^1^ The project is implemented as part of the occupational health management aiming to assist nurses and medical doctors via a 9-week training program. It is specifically designed to prevent burnout in a hospital setting and develop essential skills for navigating through stressful work-related matters, promoting overall well-being (28, 29).

## 2 Methods

### 2.1 Design

In a qualitative study we collected data in two different German hospitals through interviews with medical doctors, nurses and medical assistants.

The research question was investigated using a qualitative study design via semi-structured interviews as it allows for delving deeply into participants’ experiences, perceptions and opinions along pre-defined questions that allow for further exploration based on the participants responses (30). The study was conducted in accordance with the CORE-Q reporting guidelines to ensure explicit and comprehensive reporting of the conducted interviews (31).

### 2.2 Research team and reflexivity

The personal characteristics of the primary researcher (J.B.) are the following: J.B. is a female PhD student (MA. Of Arts, Motion and Mindfulness) (early thirties) and researcher within the LAGOM project with no formal training as a medical doctor or nurse and no work experience in the field of the evaluated target group. The researcher gained firsthand experience by observing members of the target group during the preliminary phase of the study and eventually conducted all interviews between February and December 2023. In terms of bias no intentional assumptions need to be reported other than the first experiences with the topic in the preliminary phase of the LAGOM project. The interviewees did not have further information about the interviewer other than the goals of the research and the embedment in the LAGOM project. J.B. consistently engaged in ongoing communication with the research group, reflecting on all aspects of the interview process. Furthermore, results were presented and discussed not only in the research group but also in two further qualitative research workshops. J.B. aimed to incorporate the inevitable thoughts, preconceptions and influences stemming from collaboration with these different parties and interviewees into the analysis process. This approach sought to minimize the impact of personal assumptions on the analysis.

### 2.3 Ethical considerations

An ethical approval from the Ethical Committee of Charité—Universitätsmedizin Berlin was received (EA2/110/22). The study was conducted in accordance with the Declaration of Helsinki protocols. Written informed consent was obtained from all participants. Interviewees were informed that reaching out to the research team as well as withdrawing from the study without giving reasons was ensured at all times.

### 2.4 Sample and recruitment

We initially compiled a purposive sampling (*n* = 20) with maximum variation in terms of associated department, level of expertise and years of experience in the job. This heterogeneity and sample size was intended to capture relevant differences between subgroups (study sites, novice vs. experienced professionals, outpatient vs. inpatient settings) and to identify recurring patterns within groups while facilitating comparisons across them. The decision on the number of interviews was oriented by the objective of attaining theoretical saturation, repeatedly discussed with the research group throughout the interview process (32). Furthermore, this sample size aligns with those employed in comparable studies, supporting its appropriateness for our research objectives (33, 34). Inclusion criteria was the active practice of the profession as a nurse, medical doctor or medical assistant in two German hospitals including one of the largest university hospitals in Europe offering maximum care (study site 1) and a medium-sized regional hospital specialized in rheumatology, orthopedics and integrative medicine (study site 2). Participants were recruited via in-person visits on wards (e.g., following observations), internal newsletters, solicitation via e-mail or phone and subsequent to the group discussion.

### 2.5 Data collection

The interview guide was based upon the results of the preliminary phase of the LAGOM project and was developed by an interdisciplinary team consisting of psychologists, an ecotrophologist, mind–body-medicine therapist, bio data analyst, movement scientist and medical doctors. The questions were designed flexible and with a focus on exploration to encompass a broad spectrum of themes, perspectives and perceptions related to the intervention. The predominant focus on deficits overlooked the existent resources as a pivotal dimension and foundation for preventive health initiatives which laid the groundwork for the present research. It also provided initial insights into different attitudes, perspectives and behaviors among individuals highlighting resourcefulness and motivational factors in the context of resilience and long-term health among healthcare professionals. Particularly, we identified three thematic domains that appeared pivotal for approaching the present research question: The composition and establishment of motivation and selfcare as well as the overarching theme of what sources of strength are utilized and how. By addressing the three domains we anticipated a better understanding of how employees cope with high-pressure situations and demanding working conditions. Specifically what abilities, opportunities and strategies allow them to not only endure but even thrive in their professional roles. Based on the principles of Helfferich, interview questions aimed to be “as open as possible, as structured as necessary” (35). The guide was pilot tested and sent to the interviewees prior to the interview. Interviews took place in the interviewees’ offices or in the facilities of the working group and were conducted face-to-face except for one interview that was conducted online. Besides the interviewer and the interviewee no additional person attended the interview. No repeat interviews were carried out.

The interviews were predominantly conducted in a dedicated office of the research group, ensuring a quiet, undisturbed environment with comfortable seating and soft lighting to create a calm and conducive atmosphere. Alternatively, the interview conducting researcher visited the medical professionals on ward, ensuring that the environment was equally undisturbed. The primary researcher conducted all of the interviews, with only the interviewee and the interviewer present in the room. Individuals were encouraged to articulate their experiences and perceptions freely by using a combination of open-ended, process-oriented and contextual questions. The interview ended with descriptive data: a brief job description, structure of work (e.g., in-patient care, out-patient care, intensive care), kind of shift work (if at all) and years of experience in the job. Interviews lasted between 30 and 60 min. Following each interview, notes were taken to capture thoughts and reflections. Interviews were recorded and fully transcribed via the Software audiotranskription—f4x (36). Transcripts were then revised again and corrected when necessary. Transcripts were not returned to the interviewees for correction or commenting. Visual recordings were not made.

The quotations included in this paper were translated from German into English (J.B.). The focus was on the overall content and meaning of the collected data, so a significant loss of meaning due to the translation is not to be expected.

### 2.6 Data analysis

Structuring qualitative content analysis according to Kuckartz was the guiding research methodology as it allows the combination of a-priori categories (interview guide) and text-based codes (open coding) (37). Additionally, it enables a descriptive presentation of the category system through the examination and development of causal models as well as ultimately supporting or refuting existing theories (37). Throughout the data collection process, a constant comparative method was employed, involving the continual examination of newly acquired data in relation to previously collected data to identify patterns and variations. Data was analyzed by one researcher (J.B.), however, there was an ongoing involvement of the research group to consistently reflect on and improve the coding process. An inductive-deductive approach was conducted using the software MAXQDA®. Initially, open inductive coding was employed to generate codes from the content (37). Those text-based categories were developed and later combined with the deductive categories of the interview guide to align the code system with the research question (38). Main categories derived from this process and afterwards subcodes were summarized within those main categories. In an iterative process new categories emerged, categories were renamed and subcodes were moved until they were adequately representing the data. Throughout the coding process the element of memo writing, commonly also used in Grounded Theory was applied to record thoughts and hypotheses or to distinguish between codes (39, 40). Regular discussions with other qualitative experts were conducted to enhance trustworthiness and credibility by establishing intersubjectivity. Participants did not provide feedback on the findings.

## 3 Results

After analyzing approximately 17 interviews, no entirely new themes emerged, and the final interviews supported the themes already identified. Thus, we determined that data saturation had been reached after 20 participants. The sample size achieved a balanced ratio between nurses and medical doctors and interviewees of the two different study sites. There was no expense allowance. One person was concerned about disadvantages due to the interview concerning anonymity but participated after more detailed information of the process was given. No other concerns were articulated. One of the interviewees served as an advisor in the LAGOM project, providing feedback for the developmental stages and establishment process. However, there were no apparent interferences stemming from role ambiguity as the discussion primarily revolved around personal perceptions within the work setting. Sociodemographic data regarding the participants of the study is shown in Table 1.

**Table 1:** Sociodemographic data of participants.

Characteristics		Number of respondents
Gender	Female	13
	Male	7
Age	27–63	20
	(mean: 47.3; σ = 10.86)	
Profession	Nurses	8
	Medical doctors	10
	Medical assistants	2
Institution	Study Site 1	12
	Study Site 2	8
Area of work	Orthopedic and Oncology Ward	1
	Oncology Ward	1
	Anesthesiology and Surgical Intensive Care	1
	Pediatrics and Adolescent Medicine	2
	Pediatric Oncology Ward	4
	Neonatology, Palliative Medicine	1
	Pediatric Emergency/Intensive Care	1
	Cancer Medicine	1
	Traditional European Medicine (German: Naturheilkunde)	8
Additional Function	Yes	6
	Ward management	4
	Deputy management	2
	No	14
Work structure	Stationary	15
	Ambulatory	2
	Both	3
Shift Work	Yes	7
	2-shift system	1
	3-shift system	6
	No/on-call duty	5
	No	8
Work experience after completed training (in years)	02–10	6
	11–20	7
	>20	7

**Figure 1:**
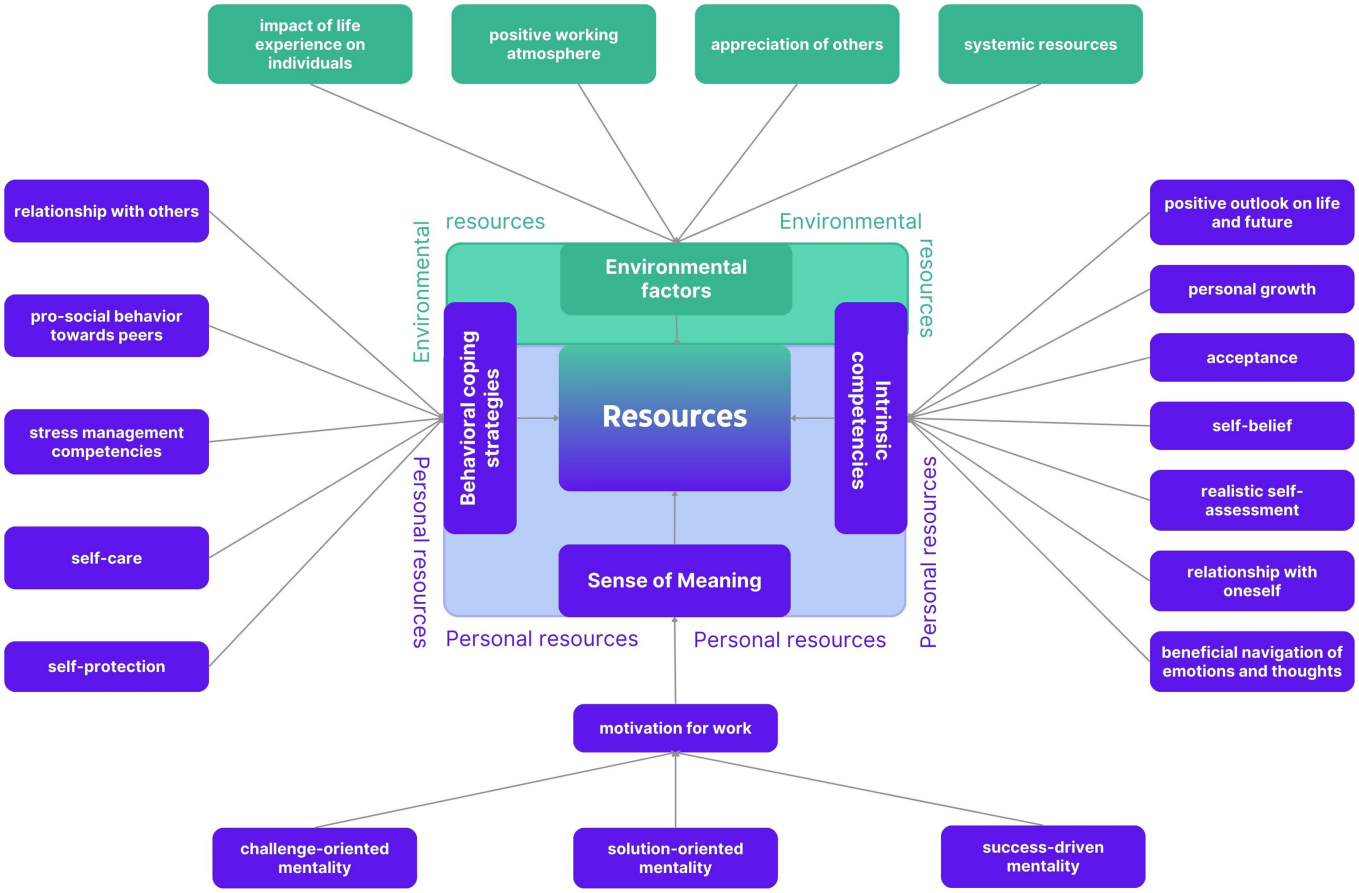
Environmental and personal resources. A selection of environmental (green) and personal resources (blue) with its 3 dimensions are shown.

Emerging from the inductive-deductive analysis, the following results are clustered into environmental resources as well as personal resources (intrinsic competencies, sense of meaning and behavioral coping strategies) revealing a spectrum of subcategories (Figure 1). Environmental resources are defined in the paper at hand as external factors the individual has no direct or sole influence on, such as positive working atmosphere and systemic resources. Personal resources are defined as innate qualities, skills and capabilities that an individual either possesses inherently or has developed over time through personal experiences or education. All four categories are described in more detail in the following sub-chapters (see also Figure 1; Tables 2–5).

**Table 2:** Categories of environmental resources with description and anchor examples.

Category	Description	Anchor example
Impact of life experiences	Influences acting on humans that are not just temporarily but sustainably shaping and reshaping the individuals’ life	“I was raised like this. I come from the former German Democratic Republic. (…) My siblings were all smaller (…) We had to do everything at home since we were children. Caring and helping my colleagues these days without questioning it is probably also inherited. It’s an inner attitude.”
Positive working atmosphere	Interplay of various factors leading to employees feeling comfortable and working well with colleagues to pursue goals	“I’ve asked my colleagues, and they say that, too: Yes, if we have had a good laugh once, then a lot has already been gained on the day.”
Appreciation of others	Others’ acknowledgement/appreciation of the hard work and achievements of individuals	“Employees must have a chance to develop and they must be seen. That is a basic principle of appreciation. If you only ever confront employees with their mistakes, this is not an environment where you provide additional energy.”
Systemic resources	Work features and conditions of the individuals’ surrounding for pursuing goals or coping with requirements and allowing a process to proceed in a targeted manner	“We have difficult conditions and, above all, we have an extremely stressful care situation. And the nursing staff are constantly working at their physical and mental limits. And of course, that also puts a strain on me, because some of them really do collapse because they can no longer cope. And there’s no improvement in sight. And we always have to close beds due to a lack of nursing staff and they have to work overtime very quickly. And that is very, very stressful.”

**Table 3:** Categories of intrinsic competencies with description and anchor examples.

Category	Description	Anchor example
Personal growth	Involvement of an individual on improving, growing and developing as a person	“This was a completely different field for me, and I really had to concentrate and learn to a point I thought I reached my limits. (…) Completely different structures, completely different ways of working. And that was stressful for me. But at the same time, I thought it was good because I mastered it and because it also gave me the experience again that I can manage it.”
Realistic self-assessment	Accurate assessment of ones’ abilities and limitations	“I am also aware that I can make a contribution and that I am well-informed, but I cannot do everything, I cannot save every child. I can do my best, but I would never believe that I am excessively competent or that I should be able to do everything, so I am fully aware that I am fallible, and I am also fully aware that my capabilities are limited somewhere.”
Self-belief	Trust in ones’ abilities to succeed at tasks and set goals	“In terms of self-awareness, I find myself in a position where I feel quite positive about myself and my abilities. I believe that I perform tasks quite well, so I have a strong and unwavering self-confidence. And I’m convinced that what I’m doing is right and I have little doubt about what I’m doing and how I’m doing it.”
Emotional regulation	Ability to exert control over one’s own emotional state (focus, emotions, impulses, behaviors)	“I can somehow quickly set aside negative feelings and problems. I do not want to spend the whole day dwelling on how terrible it is, but I want to get out of the situation, go somewhere else quickly and get involved in something else. I can still return to the situation and emphasize again but ultimately this approach helps me effectively to no longer being affected in my everyday life.”
Acceptance	Acknowledgment and agreement with the inevitability or conclusion of a situation, refraining from any attempts to alter or protest it	“But you must be able to accept that (…) I think it depends on you expressing what’s on your mind and not eating it up. So that you can simply understand for yourself and accept that it’s part of life, that sometimes things do not work out the way you plan”; “If someone has a different value system, then that’s ok.”
Relationship with oneself	Bond with oneself involving self-awareness, self-acceptance and self-care	“Through self-care, self-reflection and self-awareness you can have a good relationship with yourself. You can only deal well with yourself, when you feel yourself and know what is good for you.”
Positive outlook on life and future	Attitude reflecting a belief or expectation that the result of a particular endeavor, or outcome in general, will be favorable, positive and desirable.	“I feel comfortable in my skin, with the people who surround me and with my future prospects, then, the pressure of a challenging situation does not seem dramatic and stressful.”

**Table 4:** Categories of sense of meaning with description and anchor examples.

Category	Description	Anchor example
Challenge-oriented mentality	Willingness to engage in tasks above individuals’ baseline difficulty even potentially inducing anxiety-producing situations	“I wanted this hell. (…) When you want hell, then it can be okay. Then it can be warm and nice. Then it can be inspiring to some extent. But if you do not want it inside, then you will suffer. I see time and time again that then people suffer in the system.”
Solution-oriented mentality	Belief of finding solutions for problems by individuals’ use of critical thinking (evaluating, analyzing and deciding quickly) alongside clarity about their “why”	“Hardships pass. It’s not as hard the next day. I think that’s my attitude and I strongly defend it.”
Success-driven mentality	Casting aside thoughts of (past or future) failure to allow the mind to focus on the strategic actions necessary for success	“The question at the end is how you valorize things, what you feel is important and what you want to spend more or less time on.”

**Table 5:** Categories of behavioral coping strategies with description and anchor examples.

Category	Description	Anchor example
Relationship with others	Voluntary or involuntary interpersonal link between two or more people where thoughts, behavior or feelings are mutually related to each other	“These teams are constantly faced with challenges. Our teams are functioning very well. And challenges even fire up the team spirit because you must fight against the outside united” “Quality time with my partner and friends and visits with my family members helps me maintain a positive atmosphere. When I feel good physically and emotionally along with nurturing relationships (…) then it’s not so dramatic if a field at work is more stressful.”
Pro-social behavior towards peers	Voluntary action in which individuals pursue the intention of benefiting other people, often without having their own interests at heart	“Even my husband, who is a doctor himself, says he does not want to hear it because it’s too awful. (…) I have a colleague who is also a very, very good friend. And I think that’s something that really helps. We sometimes sit here in the evening and talk about things and work through the whole thing.”
Stress management competencies	Respectfully, responsibly managing emotions to reduce negative impacts by stress and improve ones’ physical and mental well-being	“I mean, music is meditation. So, I play quartet. That is meditation. (…) You also do something completely different. And that’s super relaxing. And otherwise cycling is also completely different. Cycling can also be meditative. I mostly cycle on the banks of the Lang Havel, the banks of the Spree, at least completely away from everything.”
self-care	routines and practices by taking an active role in protecting and improving one’s well-being and happiness	“There is an ethical principle about self-care that points out that it’s not right to give yourself up just to do justice to the patient. Because at some point you really cannot do your job properly anymore. That was a revalating moment for me, that it’s really important to look after yourself.”
self-protection	protecting and defending health and well-being of oneself from harm	“I feel that I need to protect myself a lot, particularly from the patients, to avoid being emotionally affected or attacked. I’ve noticed that I’ve become quite distant. I understand that it’s not ideal for daily interactions, but for me, it’s necessary to continue in this job.”

### 3.1 Environmental resources

Environmental resources include the possible impact of life experiences on individuals as well as (organizational) working conditions and higher-level systemic resources (Table 2). Our data shows that all these environmental resources can significantly impact how employees’ access and utilize their resources.

The way in which individual reactions to adversity at work was attributed by participants to childhood experiences and positive or negative experiences in the workplace. Negative experiences, such as feeling left down by supervisors, teammates or even patients due to unrealistic expectations to be overly of service while neglecting the necessity to also care for oneself were expressed with frustration and resignation. At the same time a positive team experience could already make the difference for the day hinting at the positive impact of a positive working atmosphere. Appreciation by colleagues (overarching hierarchy) and patients was repeatedly mentioned with no notable difference in attribution across the validating groups. The positive influences of colleagues/supervisors became especially evident in positive mentorship at the beginning of one’s career as it appeared to significantly influence the development of (work-related) self-belief: “*In the beginning of my training I had a phase where I was really well supervised and confirmed, so that you develop a kind of professional self-confidence.”* Framework conditions like autonomous workflow, co-decision making and work-redistribution due to work overload were mentioned as impactful, mainly reported by using negative examples. Systemic resources such as staffing were among the most frequently mentioned resources as deficiencies in that regard caused various difficulties such as overtime, replacement requirements and responsibility for too many patients at once. These difficulties as well as shift work aggravated the access to other resources (plannability of private events, seeking for social support, recovery routines such as leisure sports). Remarkably, in both job groups economic resources were only mentioned rarely and did not seem to have a huge impact on the perception of resourcefulness.

### 3.2 Personal resources

In addition to environmental resources, employees rely on accessing their intrinsic and actionable repertoire to respond to work demands in managing requirements.

#### 3.2.1 Intrinsic competencies

Individuals identified several factors that significantly contribute to their resilience (i.e., Table 3).

A positive attitude towards life and future emerged as a central theme, which also showed in participants’ attitudes towards adversity in the workplace: *“I’m totally optimistic, a totally optimistic person and I’m also someone who (…) tries to find the best solution with the given circumstances and does not try to get caught up on problems that I cannot change.”* This positivity also translated into a pursuit of personal growth. Individuals could view their mistakes with greater ease, focusing intensely on the lessons to be learned from them. At least after the initial phase, they refrained from dwelling on failure and instead embraced its inevitability, allowing them to move forward with acceptance. Embracing one’s own flaws and limitations, as well as those of others (e.g., patients with risky health behaviors or colleagues with differing value systems), along with daily challenges one is exposed to, formed a critical foundation for navigating adversity. Despite awareness of their own imperfections, self-belief emerged as one of the most reported resources. In a reinforcing cycle, individuals drew energy from mastering their craft to effectively help patients, receiving positive feedback on therapy outcomes and gratitude from patients. As individuals developed trust in their abilities over time and experience and the conviction of making right decisions their (work-related) self-belief and self-perception became increasingly positive. Emerging attitudes towards this aspect varied considerably across interviewees, with some individuals viewing their higher status within the professional hierarchy as an empowering and distinctive resource: *“(…) I know many people cannot and will not manage to do what I did because they do not even have the strength and, secondly, they do not have the opportunities that I have built up for myself. Nobody can just conjure them up.”* while others would emphasize humbleness: *“I would not say I’m especially right for this job. (…) That would be overestimating myself. (…) I think I’m part of something bigger.”* The last part of the quote shows that an individual’s spiritual grounding potentially fosters a sense of security. Positively assessing abilities and the self was consistently addressed as vital for perceived resilience also by categorizing oneself as equally important as patients and colleagues to keep up sustainable job performance. Individuals emphasized that cultivating a nurturing relationship with oneself serves as the foundation to successfully work (with others). Therefore, they highlighted the importance of making use of self-care strategies, daily routines and time for solitary reflection to strengthen the bond with oneself. It was repeatedly stressed that this aspect, detailed further in the behavioral coping strategies section, requires dedicated attention and time. A healthy relationship with oneself encompasses various factors, including self-protection (e.g., distancing oneself from job-related stressors), careful resource management (e.g., ensuring adequate sleep or avoiding overcommitment in leisure activities), and proactively planning and adjusting circumstances when demands exceed personal capacities. While individuals predominantly reported on their behavior responses when feeling overwhelmed such as focusing on one task at a time and disregarding intrusive thoughts, articulating cognitive patterns seemed more challenging. Nevertheless, responses reflected a variety of coping approaches as exemplified by one interviewee, a medical doctor with main responsibility regarding emergency situations:*“You have all these acute emergency situations where you simply must function in the moment, even if you are afraid yourself. Even if you are in turmoil inside, you got to keep calm and say: okay, one step at a time. (…) Pause for a moment and then you follow the next step of your plan and work through it.”* Healthcare professionals did not localize their potential to stay calm and structured in tough situations by their resistance to perceive them: *“Anyone who says they do not get nervous in rehabilitation situations, I think that’s a lie. When things get clinical, everyone’s adrenaline level rises. I have a different level of skill and security than I might have had 20 years ago, but still, when you are called to an emergency your adrenaline level always rises.”* Support pillars, potentially shaping the cognitive frameworks through which healthcare professionals navigate their emotions, include factors such as spirituality and the perceived level of meaning of their profession.

#### 3.2.2 Sense of meaning

A major resource was the ability of individuals to clearly recall and understand why they are doing what they are doing. When talking about the “why,” the sense of meaning, the majority of interviewees showed a strong sense of passion and ambition. The significance of meaning was paramount for individuals in facing challenges, including changes, even negative ones arising out of necessity. It enabled individuals to persevere through demanding phases, with a focus on the patient, fostering a more altruistic motivation: *“I would simply say understanding why you are here. And I think the focus is on the patient. That’s what it should be about.”* Patients played a crucial role in providing this sense of meaning: *“I think those are the most moving moments: when people start to see it’s not a drama, it’s manageable.”* The sense of meaning primarily served as the driving force behind continuous professional development and achieving success in conducting therapies (Table 4). Three subdivisions emerged within the category of “sense of meaning” impacting motivation: challenge-oriented mentality, success-driven mentality and solution-oriented mentality.

Emphasizing the importance of facing challenges rather than avoiding them was a recurring theme: *“I’ve always exposed myself to it. I have not fled. I’m always someone who approaches things.”* Challenge-oriented mentality also manifested in perceiving career advancement as inspiring rather than scary, with varying degrees of emphasis on ego. Meaning led to a profound determination, empowering individuals to be highly aware of stressors and still persevering forward. Success-driven mentality was evident in various situations, such as embracing mistakes as part of progress, prioritizing tasks, and maintaining clarity and structure in one’s work. Solution-oriented mentality was demonstrated by valuing situations as realistically, avoiding unnecessary drama and focusing on solutions rather than problems.

#### 3.2.3 Behavioral coping strategies

Categories of behavioral coping strategies are shown in Table 5.

Social attentiveness was perceived as an impactful resource, facilitating the establishment of nourishing social relationships with colleagues, supervisors, patients and within one’s private life. At work, pro-social behavior towards colleagues and supervisors in the field was reported as significant resource, even in comparison to interactions with spouses. Addressing concerns, sharing negative experiences, and asking for help or advice were highlighted as ways to directly improve the working atmosphere. One interviewee described the unity with patients emerging from fighting for the same goal: *“The feedback you get is energizing. This constant interaction, especially in oncology in pediatrics with its’ long-term care and accompanying. You build up a certain relationship with the patients and the parents and you gain satisfaction from it.”* Team dynamics were consistently cited as a valuable resource in navigating workplace challenges, possessing the ability to withstand even the most intense pressure phases. It was repeatedly emphasized that cultivating a supportive social environment outside of work significantly influenced resourcefulness in coping with job demands. This also accounted for the general ability to foster a balanced, enriched, enjoyable and healthy life beyond work: *“Sometimes I have the feeling that colleagues who are much more stressed in their private lives are also much more prone to burnout at work. If they are fulfilled in their private lives, then I have the feeling that they are actually not so stressed, that they are fine.”* Other examples included prioritizing early bedtime, avoiding overcommitment in one’s leisure time, or enjoying a glass of wine in the evening. Just a few interviewees reported utilizing methods to specifically target stress (for example meditation or yoga). The majority of employees reported engaging in actions that inadvertently helped manage stress, often realizing their stress-reducing effects only upon reflection. Numerous hobbies were cited to function as valuable resources, with sports and music being the most frequently mentioned areas. Allocating time for solitary reflection without external distraction was viewed as a crucial resource for replenishing energy levels. In addition to time for self-reflection, many employees discussed the importance of self-empowerment, with some even seeking guidance from personal coaches: *“The best thing that happened to me was being able to undergo coaching for a period of time. It truly provided me with a different perspective. (…) Professional coaching is much more personal and has a profound impact on one’s personal growth.”* An array of self-care strategies was discussed, including taking breaks (also smoking breaks during shifts), being aware of and ensuring basic needs are met (such as bathroom breaks and staying hydrated), putting effort into one’s appearance and frequently visiting doctors for health check-ups. Furthermore, engaging in energy work, spirituality or using EFT (emotional freedom techniques) was mentioned as resource by some participants. Interviewees emphasized the importance of actively designing their lives to cultivate supportive environments. They also emphasized the use of self-protection strategies, such as clear communication with spouses about the inevitability of fulfilling job demands, as integral to them. Self-protection was highlighted as a resource repeatedly, yet it also underscores the conflict between its functionality and overall health-promoting nature.

### 3.3 Observed interrelations between categories

In addition to presenting results based on the participants’ narratives as above, the interviews revealed overarching connections and notable features. The richness of the information emerged not only from the content of words but also from the way in which it was conveyed. The immense pressure of some interviewees was visible in emotional conversations with some even tearing up or showing their frustration by anger. In contrast, others appeared calm and collected even though the pressure to perform well seemed particularly high from an outer perspective. Therefore, as the number of interviews increased, interrelations as well as the essence of some topics became clearer, and will be described in the following section allowing for including some observations and perceptions of the interviewer.

Initially, it became apparent that subcategories influence each other with expressions on one subcategory often enhancing accessibility to others. This was not only notable within subcategories of a single category but across different categories as well. For example, individuals who frequently engaged in realistic self-assessment (intrinsic competency), were much more likely to apply pro-active self-protection methods (behavioral coping strategies) such as clearly vocalizing limits and capabilities. Another example is that individuals with a better reported relationship with themselves seemed to navigate their emotions and thoughts more efficiently. Moreover, the degree to which intrinsic competencies are cultivated appears to significantly influence an individual’s ability to navigate emotions and thoughts. An important question arises as to what extent the perception of security through spiritual grounding positively influences this. It may even alleviate personal burdens, such as the fear of making mistakes by anchoring therapeutical success not solely in their professional performance but in a greater perspective. Furthermore, achieving high work performance in healthcare seems to depend more on professionals’ ability to manage difficult emotions rather than their resistance to recognizing the potential finality of making mistakes, as well as their level of experience. The disadvantage of a rather passive attitude was evident in individuals focusing on systemic errors with high levels of frustration. These individuals did not see a (better) alternative than continuing working in their position, highlighting the importance of the cycle of perceived self-efficacy, which leads to activity when high or passivity when low. This passivity may stem from adopting the high expectations of the environment as their own, leading to high expectations towards the system as a positive resource. When these expectations are unfulfilled, it causes a significant loss of resourcefulness. Our data indicates that the better intrinsic competencies such as self-belief, positive self-assessment, and relationship with oneself the less the focus and perceived dependance on environmental resources. These individuals recognized the inefficiencies of the system nonetheless and often perceived the disadvantageous effects on a personal level but without attaching their dependance on them. Limiting factors that aggravate access to other resources potentially facilitate unwanted stress management behaviors, such as taking more breaks to smoke or stressed-induced eating. Therefore, beneficial systemic and organizational resources potentially create a nourishing link between job demands and adaptive self-regulation, mitigating the negative influence of job demands on maladaptive self-regulation. Lastly, the proactive implementation of knowledge into action, even when recognizing the necessity and benefits of health-promoting behaviors, proved to be considerably challenging. Unfortunately, frequently reported facilitating factors included health problems or significant disruptions like experiences of burnout, which forced individuals to enact change.

## 4 Discussion

In this publication we offered insights into the resources health care professionals utilize in the face of work strains. Our findings highlight a broad spectrum of resources categorizable into personal resources with its three categories intrinsic competencies (1), sense of meaning (2), and behavioral coping strategies (3) as well as environmental resources. Our data shows that resourcefulness is deeply personal, yet a set of resources was repeatedly reported, underscoring their consistent importance. Our aim was not to construct a definitive profile of a highly resilient individual or to pinpoint the precise resources that foster individual resilience. As our objective rather aimed to assess the resources that are employed by healthcare professionals in managing daily challenges on a broader level, we are aware of the higher complexity to what specifically contributes to individual resourcefulness. A glimpse of that complexity was shown by highly varying degrees of importance of different resources across participants. It even seemed that the expression of just a few subcategories could make the difference in successfully managing work demands sustainably.

Some of the sub-categories are among the most consistently reported predictors of resilience-promoting personality variables. These include supportive resources, emotional regulation strategies and searching for meaning, alongside others according to personality traits that constitute a motivational basis for adaptive responses (41, 42). Furthermore, optimism, as demonstrated in our results in *positive outlook on life and future* encourages proactive engagement and a readiness to strive for a positive future (43). Beyond that, optimism has been shown to interact with other traits such as challenge orientation (44). This is in alignment with our observation of interrelations in between categories, suggesting that approaching one aspect will have a multiplier effect on other resources. Other interrelations became apparent. For instance, our findings suggest that individuals with lower self-efficacy in the face of job strain tend to show lower disposition to apply adaptive self-regulation strategies, whereas those with a sense of control demonstrate pro-active approaches. This observation is in alignment with the *Job Demands-Resources theory* by Bakker and De Vries that describes the effects of job strain leading to avoidance coping and self-undermining. In a vicious cycle this again leads to more job demands and more job strain ultimately resulting in the loss of personal and job resources (21). Employees equipped with essential personal resources proactively prevent job burnout by implementing their stable characteristics and abilities (ibid.). Questions remain regarding the optimal combination (what set of resources) and distribution of resources (e.g., is the strong manifestation of one resource as valuable as an equal resourcefulness distributed across many resources).

Numerous studies attempted to pinpoint a set of resources leading to resilience or identify specific personality types as resilient. However, these studies often employ resilience questionnaires, whose validity is to be questioned due to their multifaceted nature, highlighting the potential benefits of adopting a broader perspective (20, 23). In a more simplified version for example, our data pointed to the important role of perception of stress as well as the conjunction between intrinsic competencies and its implementation (behavioral coping strategies) which accounts for self-efficacy as main moderating factor in resource accessibility. Parallelly, Bonanno (20) suggested flexible self-regulation as mechanism that underlies resilience, which we in this context may also refer to as accessibility to resources, considering various situations and different points in time (20). The contextual sensitivity (step 1) to a stress inducing situation (“what is happening?,” “what do I need to do?”) as well as the repertoire (step 2) (“what am I able to do?”) and ability to successfully monitor feedback (step 3) represent the *flexibility sequence* considering these two aspects (ibid.). A once successfully employed flexible sequence may therefore not work at another point in time or/and in other situations no matter how similarly adopted the flexibility sequence that proved successful before. As the so-called *resilience paradox* describes the inability of a single factor or collective sum of resilience factors to fully explain the complex nature of resilience, the access to the set of beneficial resources when employed via a beneficial adaptive coping strategy may therefore depend on further determinants (20). Furthermore, a gap in between self-reports and actual efficiency aggravates the definitive allocation of resources. As shown by study results targeting resilience, that indicated that people scoring high on a resilience profile, as determined by a personality index, exhibited elevated stress levels similar to those scoring low on the resilience profile (23, 24). For example, in one study participants (students) conducted the Trier Social Stress Test, were asked to give an oral presentation in front of experts with little preparation time and were videotaped and recorded (24). By reviewing the videotapes as well as markers of physical arousal, resilient students showed increased stress reactions just as much. The results of this study have led to the conclusion that the concept of resilience as a personality trait may be nothing more than a self-deception artifact as resilient participants were only better equipped in their self-report and not in their actual behavior (25). Addressing this discrepancy requires incorporating objective measures alongside subjective self-reports. Future studies could add observational methods and physiological assessments to provide a more holistic perspective on the role of resilience in workplace well-being. The unique interplay between resource abundance and its application in a specific situation and at a particular time underscores the complexity of the subject under analysis. Further challenges to identify resilience based on character traits or self-reported sets of resources, reveal the need for a broader perspective to explore further avenues of inquiry.

One approach could be the analysis of underlying motivations to withstand adversity such as the goal of individuals about what life they want to lead (22). As suggested in recent research, the hedonic well-being approach to lead a *happy life* characterized by safety, stability, comfort and pleasantness is to be distinguished from the eudemonic approach of leading a *meaningful life* characterized by purpose, service, devotion and sacrifice as well as from the *psychologically rich life* characterized by novelty, variety and interest (22). Our data revealed resources in all those characterizations and strongly indicates that a sense of meaning and purpose may enhance resourcefulness in the face of adversity, which has also been shown in prior research (45). Therefore, depending on the different aspirations in life, individuals may develop and utilize a specific set of resources accordingly and withstand different burden better than others without negative consequences. A person following the aspiration of leading a meaningful life thus may be better equipped to endure hardships and sacrificing physical and psychological comfort unharmed due to devotion and purpose than people who follow the aspiration of leading a happy life. People following a meaningful life as a result may spend less time in a comfortable, pleasant, and stable space as their perceived purpose and meaningfulness allows them to stay in longer and more intense discomfort than others. Our findings consistently highlight meaning and purpose as crucial components identified by the surveyed job groups when identifying resources. This underscores the notion that these factors are highly valued and may serve as a profound resource for coping with the significant job burden, they face. As the differing aspirations in life may determine how individuals interpret and respond to workplace challenges, future research should examine the interplay between personal values and coping strategies. However, environmental influencing factors like overtime and shift work may limit access to certain valuable resources (e.g., social support of beloved ones), potentially exacerbating reliance on maladaptive coping mechanisms.

In examining resources, self-care behaviors and coping mechanisms among healthcare professionals, valuable insights could be gained into how healthcare professionals navigate adversity in their daily lives. However, there were limitations as presented in the following section.

### 4.1 Limitations

A notable aspect of the applied study design was that all interviews were conducted solely by the first author. This approach ensures consistency throughout the interview process while it also introduces the possibility of interviewer bias, as the interpretation and framing of questions might reflect the perspective of a single individual. To midigate this bias interviews were reflected post-conduction in the research team. While the interview conducting researcher had no shared professional experience, framing of questions or interpretation of responses potentially limit the scope of insights. Yet the firsthand exposure to the field in a preliminary phase of the study may have facilitated the development of a deeper understanding of the healthcare professional’s experiences. Since the interviews were conducted in German, effort was made to maintain accuracy and fidelity during the translation process by involving a native English speaker though nuances may have been lost or altered. As our examination focused solely on self-reported resources, limitations arise in understanding resource accessibility. Furthermore, the study lacks analysis of the health-promoting effects of identified resources, raising questions about potentially harmful (though effective) behaviors such as smoking breaks. Additionally, the paper overlooks how hospital work environments and the education leading up to it shape individuals’ stress management strategies as well as it does not take into consideration if the work environment attracts specific personality types. Not distinguishing between nurses and medical doctors is a major limitation, given the different work responsibilities, opportunities, and the differing societal statuses. Moreover, despite aiming for maximum variety, the high proportion of female nurses and lack of particular consideration of foreign workers limit the study’s scope and applicability of the findings. Regarding the development of categories, a clear separation was difficult due to subcategory as well as inter-category relations. The process of presenting distinct categories was further aggravated due to notable influences in between them. For example, *challenge-oriented mentality* appeared to be linked to *self-belief* as well as *positive working atmosphere* and/or *appreciation of others* as these factors played a crucial role in the positive anticipation of pay-off and therefore motivation to succeed at employees’ craft.

Results suggest that the more individuals relied on their personal resources, the less they relied on less-controllable environmental resources. The perception of being in control furthermore seemed to have a positive influence on the success of utilizing coping mechanisms to deal with demands and challenges, whereas focusing and relying on environmental factors (e.g., systemic resources) led to dis-contentment and the feeling of victimhood of circumstances. Future research should further explore this relationship by developing targeted interventions that enhance the perception of control among healthcare professionals.

Providing a work environment on a broader level that holds a space in which staff can experience inspiration and motivation to thrive at work has been disregarded in recent decades. At the same time employees may have learned powerlessness due to the systemic burden which may has resulted in passivity instead of proactively improving their situation with available options. However, the systemic aspect is indispensable recognizing that individual resilience can only alleviate the overall situation to a limited extent. Impactful change occurs most robustly through a collaborative effort that integrates both top-down and bottom-up strategies.

Implications for occupational health promotion in the hospital setting.

The above highlights the significance for the research at hand, which provides quite a few starting points on the institutional and individual level besides the systemic necessity of ensuring adequate staffing levels and more broadly focusing less on profitability. The following list shows pathways to promote resources of healthcare professionals on an institutional and individual level.

Pathways to promote resources of healthcare professionals on an institutional level:

- Providing a close supervision by adequate staff to empower individuals in the initial phase of their career
- Investing in excellent team dynamics
- Structural implementation of conflict management
- Providing an open ear policy and address stressors
- Ensuring autonomous workflow and co-decision making when possible
- Frequently monitoring work overload and initiating work-redistribution
- Framework conditions that allow self-care within the work setting without having individuals feel like they can only do so neglecting social responsibilities or letting down colleagues and patients

Pathways to promote resources of healthcare professionals on an individual level:

- Developing a clear why at the initial phase of the career
- Making use of available support offers
- Using one’s opportunities to pro-actively influence the work environment
- Asking for and using opportunities to engage in further training
- Thoughtful resource management in leisure time
- Building healthy routines

## 5 Conclusion

To work in the hospital setting offers a unique opportunity for a personally enriching work life, providing a strong foundation for experiencing a deep sense of purpose as well as opportunities for personal growth. Individuals use a variety of resources and their ability to withstand workplace challenges and overcome adversity appears to be enhanced when their personal values align with those provided by their work environment. Feeling a strong sense of meaning in their work and a desire to lead a purposeful life, therefore, seem to improve the capacity to cope with work-related adversity. Individuals displayed a range of intrinsic competencies and behavioral coping strategies. The interconnection between categories—especially aligning inner experience (intrinsic competencies) with behavior (behavioral coping strategies)—potentially empowers individuals to effectively manage stress and enables the realization of complex medical diagnostics and therapies sustainably. Moving from a problem-focused attitude towards innovative pathways, it is crucial to emphasize that this research does not overlook the imperative for systemic change in the hospital sector. Providing a work environment on a broader level that holds a space in which staff can experience inspiration and motivation requires both bottom-up (individual) but especially top-down (institutional and systemic) initiatives.

## Data Availability

The datasets presented in this article are not readily available because the data collected in this study contain sensitive information, particularly interview data with health care professionals. Due to ethical and data protection regulations, sharing or publicly storing these data is not possible, even after pseudonymization. Requests to access the datasets should be directed to julia.berschick@charite.de.

## Footnotes

^1^Charité-Universitätsmedizin-Berlin. 2024. Available online at: https://kinderonkologie.charite.de/forschung/ag_seifert/lagom_projekt/
